# Comparisons Between Recruitment and Retention Strategies for Two Online RCTs

**DOI:** 10.1093/geroni/igab046.314

**Published:** 2021-12-17

**Authors:** Britney Webster, Alexandra Jeanblanc, Gregory Smith, Frank Infurna

**Affiliations:** 1 Kent State University, Kent, Ohio, United States; 2 Case Western Reserve University, Cleveland, Ohio, United States; 3 Arizona State University, Tempe, Arizona, United States

## Abstract

Custodial grandfamilies (CGF) comprise a small, diverse group of the US population which can make samples difficult to recruit and retain. Two online RCT studies (S1 & S2) for CGF used a variety of recruitment strategies with varying success. S1, for grandmothers (GM) only, successfully recruited from Facebook (47.95%) and community flyers (17.73%). S2, dyadic study for GM and adolescent grandchildren (AGC), recruited through emails to high school counselors (43.29%) and community (30.94%) and professional (17.13%) kinship support organizations. The advantages of online RCTs for hard-to-reach populations include expedited administration, buffering against social distancing, nationwide enrollment (S1-42 states; S2-43 states), and generalizability of findings. Challenges of online RCTs are establishing rapport and building trust with participants who are not comfortable with technology and designing screenings to identify false participants. Overall, these studies highlight the advantages of an online RCT, especially for hard-to-reach populations like custodial grandfamilies.

